# Development of avian influenza virus H5 DNA vaccine and MDP-1 gene of *Mycobacterium bovis *as genetic adjuvant

**DOI:** 10.1186/1479-0556-8-4

**Published:** 2010-05-24

**Authors:** Babak Jalilian, Abdul Rahman Omar, Mohd Hair Bejo, Noorjahan Banu Alitheen, Mehdi Rasoli, Sohkichi Matsumoto

**Affiliations:** 1 Institute of Bioscience, University Putra Malaysia, Serdang 43400, Selangor, Malaysia; 2 Faculty of Veterinary Medicine, University Putra Malaysia, Serdang 43400, Selangor, Malaysia; 3 Faculty of Biotechnology and Biomedical Sciences, University Putra Malaysia, Serdang 43400, Selangor, Malaysia; 4 Department of Bacteriology, Osaka City University Graduate School of Medicine, 1-4-3 Asahi-machi, Abeno-ku, Osaka 545-8585, Japan

## Abstract

**Background:**

Studies have shown that DNA vaccines can induce protective immunity, which demonstrated the high potential of DNA vaccines as an alternative to inactivated vaccines. Vaccines are frequently formulated with adjuvants to improve their release, delivery and presentation to the host immune system.

**Methods:**

The H5 gene of H5N1 virus (A/Ck/Malaysia/5858/04) was cloned separately into pcDNA3.1 + vector. The immunogenicity of the cloned H5 DNA vaccine was tested on SPF chickens using two different approaches. First approach was using H5 DNA vaccine (pcDNA3.1/H5) and the second was using H5 DNA vaccine in addition to the pcDNA3.1/MDP1 vaccine. Ten days old chickens inoculated three times with two weeks intervals. The spleen and muscle samples from chickens immunized with H5 (pcDNA3.1/H5) and H5 + MDP1 (pcDNA3.1/H5 + pcDNA3.1/MDP1) vaccines were collected after sacrificing the chickens and successfully expressed H5 and MDP1 RNA transcripts. The sera of immunized chickens were collected prior to first immunization and every week after immunization; and analyzed using enzyme-linked immunosorbent assay (ELISA) and hemagglutination inhibition (HI) test.

**Results:**

Results of competitive ELISA showed successful antibody responses two weeks post immunization. The HI test showed an increased in antibody titers during the course of experiment in group immunized with H5 and H5 + MDP1 vaccines. The result showed that the constructed DNA vaccines were able to produce detectable antibody titer in which the group immunized with H5 + MDP1 vaccine produced higher antibody comparing to H5 vaccine alone.

**Conclusions:**

This study shows for the first time the usefulness of MDP1 as a genetic adjuvant for H5 DNA vaccine.

## Background

Influenza virus can cause an acute, highly transmittable respiratory disease, which can result in high morbidity and mortality in both human and animals [1]. The 1997 Hong Kong outbreak of highly pathogenic avian influenza virus (HPAI)-H5N1 showed that avian influenza is a potential threat to human and is believed to be transmitted from infected birds [2]. The Hong Kong outbreak of avian influenza H5N1 was controlled by slaughtering 1.5 million chickens, which cost more than 245 million dollars in a single month. Therefore, antivirals and vaccines seem to be a more prospective solution to control the outbreaks of avian influenza virus [2].

Currently, whole virus inactivated vaccines containing HA as the main component, are the common vaccines to prevent avian influenza. However, these vaccines require large numbers of specific-pathogen-free embryonated chicken eggs and about 6 months to propagate the viruses [2]. On the other hand, this is not an ideal method to produce inactivated vaccine for highly pathogenic strains, as the embryos are killed shortly after propagation and require a high level of biosecurity to handle [3]. Commercial vaccines have been successful in producing protective immunity against infections by homologous virus but failed in preventing the outbreaks of heterologous virus and occasionally been reported as a possible cause of re-emerging outbreaks [2]. The commercially available vaccines against H5N1 are inactivated whole virus vaccine and fowlpox virus vaccine expressing the H5 gene [4]. Moreover, various recombinant vaccines against avian influenza H5N1 virus which are able to induce different levels of protective immunity, such as DNA plasmid-based vaccine, baculovirus recombinant H5 vaccine, and reverse genetic H5 vaccine have been examined experimentally [5-7].

Concurrent studies have revealed that DNA vaccines encoding HA of influenza A virus can result in the development of protective immune response against influenza virus challenge in animals [8,9]. In most cases, two or three doses of naked plasmid DNA are required to induce immune response to the pathogen [10,11]. Nevertheless, other studies have shown that a single dose of DNA vaccine can trigger protective immunity, which demonstrated the high potential of DNA vaccines as an alternative to inactivated vaccines [12,13]. Recently, we have showed that the fusion of ESAT-6 of *Mycobacterium tuberculosis *to H5 DNA vaccine are able to improve the antibody titer of chickens against AIV showing the flexibility of modifying the efficacy of DNA vaccine [14].

Mycobacterial DNA binding protein 1 (MDP1) is a main cellular protein produced by *Mycobacterium bovis*. The protein has both nucleic acid binding activity and macro-molecular bio-synthesis inhibitory properties that play key role in modulating bacterial growth [15]. Prabhakar *et al.*, in 1998, revealed that DNA binding proteins (orthologus with MDP1) may act as an immunodominant antigen which stimulates cellular and humoral responses presumably through TLR9 dependent pathway production of proinflammatory cytokines [16,17] and the induction of IFN-γ production [18,19].

Hence, MDP1 may play an important role as a potential adjuvant to boost the immunotherapeutic effects of DNA vaccines.

## Methods

### Construction of recombinant DNA plasmids

Construction of eukaryotic expression plasmids were performed by separately cloning the HA gene of H5N1 AIV (A/chicken/H5N1/5858/2004) and MDP1 gene of *Mycobacterium bovis *into pcDNA3.1 + vectors (Invitrogen^®^, USA). The full length H5 gene (1707 bp) was amplified from pCR2.1/H5 (kindly provided by Nurul Hidayah, Biologics Lab, University Putra Malaysia) using forward and reverse primers with *Hind*III and *Bam*HI sites, respectively (Table 1). The MDP1 gene which was provided by Prof. Dr. Sohkichi Matsumoto from Department of Bacteriology, Osaka City University Graduate School of Medicine, Osaka, Japan; was amplified from pcDNA3.1/MDP1 using forward and reverse primers with *Hind*III and *Bam*HI sites, respectively (Table 1). The amplified genes of H5 and MDP1 were digested with *Hind*III and *Bam*HI to generate cohesive ends for cloning into pcDNA3.1. The digested products were purified by electrophoresis and ligated into pcDNA3.1 using T4 ligation system (Vivantis^®^, Malaysia). The constructed plasmids were transformed into competent *Escherichia coli *Top10F' and cultured overnight for further application.

**Table 1:** Primers designed for amplification of H5 and MDP1 genes.

Name	Type	Sequence (5' to 3')
H5	F	CCC CAA GCT TAT GGA GAA AAT AGT GCT T
	
	R	CCC GGA TCC AAT GCA AAT TCT GCA TTG TAA

MDP1	F	CCC AAG CTT ATG AAC AAA GCA GAG CTC
	
	R	AAA GGA TCC CTA TTT GCG ACC CCG

F: Forward, R: Reverse

Underlined sequences are the restriction enzyme sites

A PCR screening approach was used to detect the presence of the desired ligated DNA on the recombinant plasmids using the same forward and reverse primers which were used in amplifying H5 and MDP1 genes, respectively (Table 1). The selected recombinant clones were further confirmed by restriction enzyme (RE) analysis and sequencing. Sequencing was carried out using a 48 capillary 3730 DNA Analyzer^® ^(Applied Biosystems^®^, USA) with both the aforementioned primers for H5 and MDP1 genes as well as the T7 promoter and BGH reverse universal primers.

### Transfection

Cell culture technology was used to test the *in vitro *expression of the genes of interest from the cloned plasmid. Vero cells (passage 71) were maintained in DMEM media (Gibco^®^, England) containing 10% bovine fetal serum (BFS) (HyClone^®^) and 1% ampicillin (50 μg/ml) (Biobasic Inc. ^®^). The day before transfection, cells were sub-cultured in a 6-well plate to have 80% confluency on the day of transfection. Transfection of each plasmid was performed using Lipofectamine™ 2000 according to the manufacturer's protocol in which 100 μl of Opti-MEM^® ^was mixed with 1 μg of desired plasmid. The plate was incubated and the cells were harvested at 24, 48 and 72 hours post transfection for the detection of protein expression using SDS-PAGE and Western blotting assays.

### Western blotting

Prior to Western blotting, a SDS-PAGE gel was run using a BenchMark™ pre-stained protein ladder (Invitrogen^®^, USA). A BioRad^® ^transblot machine was then used to transfer the expressed proteins from the SDS-PAGE gel to a nitrocellulose membrane using a constant current of 15 volt and 60 mA for 90 minutes. To detect the expression of different proteins, the membrane was incubated with different primary antibodies. Detection of H5 protein were performed using rabbit polyclonal antibody against AIV hemagglutinin A/chicken/Jilin/9/2004 (H5N1) (diluted 1:4000) (AbCam^®^, USA), whilst expression of MDP1 with MDP1 monoclonal antibody (1:200) which was provided by Prof. Dr. Sohkichi Matsumoto. The membranes were incubated with primary antibody solution for 1.5 hours at room temperature. The membrane was then incubated in anti-rabbit secondary antibody solution (diluted 1:4000) (AbCam^®^, USA), for 45 minutes at room temperature on a rotary shaker. Finally, the membrane was incubated in 5 ml of chromogenic solution (BCIP/NBT substrate for alkaline phosphatase) until the bands appeared.

### Immunization of the chickens with constructed DNA vaccines

Briefly, agar plate containing 50 μg/ml ampicillin was cultured using the glycerol stock of target plasmid overnight at 37°C. A single colony from the plate was cultured in 5 ml of LB broth containing 50 μg/ml ampicillin at 37°C for 8 hours with vigorous shaking. Two ml of the culture was inoculated in 200 ml of LB broth with 50 μg/ml ampicillin and shaked vigorously at 37°C for 15 hours. The bacterial pellet was obtained by centrifuging the culture in 200 ml centrifuge tubes at 6000 × g for 15 minutes at 4°C. The plasmids were then extracted using EndoFree^®^Plasmid Mega Kit (Qiagen^®^, Germany). The concentration and purity of the plasmid were determined using BioRad smart spec™ 3000 spectrophotometer. The solution was adjusted to 1 μg/μl and stored at -30°C for immunization trials. Specific-pathogen-free white Leghorn layer chickens were kept in separate cages for each group and fed twice a day using commercial chicken pellet while water was provided ad libitum. Ten days old chickens were tagged using metal wing tags and divided into five different groups with nine chickens in each group, namely H5, H5 + MDP1, pcDNA3.1 +, PBS and control. The last three groups were the different categories of negative control groups consisting of chickens immunized with parental plasmid alone, saline and left unimmunized, respectively. Ten days old chickens were immunized with 100 μg of purified plasmid via intramuscular route on the right pectoral muscle. The chickens in H5 + MDP1 group were immunized with 100 μg of H5 vaccine on the right and 100 μg of MDP1 vaccine on the left pectoral muscles. Two booster immunizations were administered within two weeks intervals after the first immunization. The first bleeding was performed via wing vein prior to the first vaccination and repeated every week post immunization for 5 weeks. The immunization trials followed internationally recognized guidelines and approved by animal care and use committee (Ref No. UPM/FPV/PS/3.2.1.551/AUP-R51) at the Faculty of Veterinary Medicine, University Putra Malaysia.

### Enzyme-linked immunosorbent assay (ELISA)

The sera derived from immunized and control chickens were subjected to a competitive ELISA test using a qualitative ELISA kit (AniGene^®^, Korea). Briefly, the plates were pre-coated with recombinant H5 avian influenza virus antigen (Anigen^® ^H5 AIV Ag). Fifty μl of serum and 50 μl of Mab-HRP were added to the wells and incubated for 90 minutes at 37°C. The wells were then aspirated and washed several times to remove the unbounded material. Following that the substrate solution was added to the wells and incubated at room temperature for one hour. The reaction was stopped by adding the stop solution and a spectrophotometer (450 nm and 620 nm) were used to read the colorimetric results. The percent inhibition (PI) value was calculated using, PI value = [1 - (OD sample/mean OD negative)] × 100 formula in which the samples with PI value of 50 and more were considered positive.

### Hemagglutination inhibition assay (HI)

The HI test was performed using the serum samples obtained from chickens immunized with different DNA vaccines. A low pathogenic H5 AIV, [A/MY/Duck/8443/04 (H5N2)] inactivated in 2-bromoethylenne hydrobromide, titrated at 4 HA/25 μl were used in the test. Briefly, 50 μl of serum was added to the first well and serially diluted to the 11^th ^well (1:2 to 1:1024). The diluted serum was then incubated with 25 μl of inactivated H5N2 virus at room temperature for 20 minutes. Twenty five μl of 0.65% washed chicken RBC was added to all the wells in plate and incubated for 30 minutes. The test results were read on a plate reader apparatus and statistically analyzed using repeated measure ANOVA. The sequence analysis of the H5 of the H5N2 showed more than 87% similar with the H5 of H5N1 in use (data not shown).

### Reverse transcription-polymerase chain reaction

The chickens were sacrificed one week after second booster. The spleen and muscle samples from the injection site were harvested and used for RT-PCR. Total RNA extraction of the samples was performed using Trizol^® ^as described by the manufacturer (Tri Reagent^®^, Life Technologies, USA). The extracted RNA was subjected to RT-PCR using a commercial RT-PCR kit (Promega^®^, USA). The PCR mixture and condition were carried out as described previously by Oveissi *et al. *with slight modifications [14]. The extracted RNA was subjected to RT-PCR using a commercial RT-PCR kit (Promega^®^, USA). AMV Reverse Transcriptase High Conc. (15 units/mg) was used to reverse transcribe 2 μg of respective RNA in the presence of dNTP's (250 mM), reverse transcriptase buffer (10 mMTris-HCl, 50 mMKCl, 0.1% TritonR-X-100), oligo dT primers (0.5 mg) and RNasin Ribonuclease inhibitor (1 unit/ml). The amplified product was run in 2.5% agarose gel at 70 volt for 45 minutes. The RNA preparations were standardized by RT-PCR for β-Actin and were free from DNA contamination evaluated by the lack of signal following non-reverse transcribed RNA using the same samples and set of primers (Table 2).

**Table 2:** Primers for RT-PCR amplification of H5, MDP1 and β actin genes

Name	Type	Sequence (5' to 3')	Length (bp)	Product
H5	F	TCCAAAGTAAACGGGCAAAG	20	141 bp
		
	R	TGYTGAGTCCCCTTTCTTGA	20	

MDP1	F	TCACACAGAAATTGGGCTCGGA	22	196 bp
		
	R	GACGTCGGCTTCACCTTTACTG	22	

β actin	F	GCAGGAGTACGATGAATC	18	140 bp
		
	R	AAATAAAGCCATGCCAATC	19	

## Results

### Cloning of the H5 and MDP1 gene into the pcDNA3.1 + vector

The constructed pcDNA3.1/H5 and pcDNA3.1/MDP1 were transformed into TOP10F' *Escherichia coli *and the positive clones were screened using PCR, RE analysis and sequencing. Digestion with *Bam*HI and *Hind*III confirmed the presence of H5 and MDP1 based on the detection of the bands of the expected sizes (data not shown). The sequencing results were checked with the original sequence of the genes deposited in the GeneBank database using the Blast program of National Institute of Biotechnology Information (NCBI).

### Transient expression of the recombinant plasmids in Vero cells

**Figure 1:**
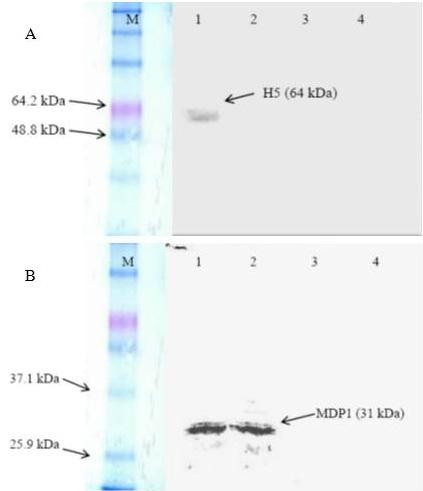
**Western blot analysis of Vero cells transfected with (a), H5 and (b) MDP1 genes**. Expression of H5 protein (product of ~ 64 kDa) was detected 72 hours after transfection while the expression of MDP1 protein (product of ~ 31 kDa) was detected 48 and 72 hours after transfection. (A) Lane M is Prestained™ protein marker (Invitrogen^®^, USA); Lane 1, 2 and 3 are Vero cells harvested 72, 48 and 24 hours, respectively, after transfection of pcDNA3.1 + with H5; Lane 4 is the non-transfected Vero cells. (B) Lane M is Prestained™ protein marker (Invitrogen^®^, USA); Lane 1, 2 and 3 are Vero cells harvested 72, 48 and 24 hours, respectively, after transfection of pcDNA3.1 + with MDP1; Lane 4 is the non-transfected Vero cells.

The expressions of H5 and MDP1 genes in Vero cells were analyzed by SDS-PAGE and Western blot. In Western blot analysis, expressed proteins for H5 (64 kDa) (Figure 1A) were detected 72 hours after transfection while the expressed protein for MDP1 (31 kDa) (Figure 1B) were successfully detected 48 and 72 hours after transfection.

### Enzyme-linked immunosorbent assay

The AIV H5 antibody was successfully detected by a competitive ELISA starting at 21 days post immunization on two out of nine chickens immunized with H5 + MDP1 vaccine. At 42 days post immunization eight out of nine chickens in the above group demonstrated antibody responses against AIV (Table 3). However, the number of chickens with antibody responses in group immunized with H5 alone is lower compared to chickens immunized with H5 + MDP1. Only five out of nine chickens in the H5 group demonstrated antibody responses at day 42 post immunization, as shown in Table 3. Chickens from the negative control groups (pcDNA3.1/MDP1, pcDNA3.1 and left unimmunized) failed to demonstrate detectable antibody response (Table 3).

**Table 3:** Detection of H5 AIV antibody from serum samples using ELISA.

Group	Days post immunization					
	
	0	7	14	21	28	35
pcDNA3.1/H5	0/9*	0/9	0/9	2/9	3/9	5/9

pcDNA3.1/H5 + pcDNA3.1/MDP1	0/9	0/9	2/9	4/9	5/9	8/9

pcDNA3.1/MDP1	0/9	0/9	0/9	0/9	0/9	0/9

pcDNA3.1 +	0/9	0/9	0/9	0/9	0/9	0/9

Negative control	0/9	0/9	0/9	0/9	0/9	0/9

* The ratio of positive treatments to the inoculated chickens

### Hemagglutination inhibition assay

The HI titer of the serum samples two weeks after the first vaccination was zero or very low (≤ 2). All the chickens in the group immunized with H5 vaccine and H5 + MDP1 vaccines showed HI antibody titer at day 21 post immunization (Table 4). The chickens in group immunized with H5 + MDP1 vaccines have slightly higher mean HI titer (3.33 ± 2.42) compared to chickens in the group immunized with H5 vaccine alone (2.33 ± 0.82). The increase in the HI titers was recorded in both groups at two weeks after the first booster and one week after the second booster. The mean HI titers from chickens immunized with H5 + MDP1 vaccines were higher than mean HI titers recorded from chickens immunized with H5 vaccine. However, the HI titers for both groups never exceeded 16. The highest average antibody titers were detected one week after the second booster, at day 35 post immunization of 13.33 ± 4.13 in chickens immunized with H5 + MDP1 vaccines, as shown in Table 4. Thus, higher antibody titer were observed in chickens immunized with H5 + MDP1 vaccines, compared to chickens immunized with H5 vaccine at day 14, 21, 28 and 35 post vaccination (Table 4). However, the HI titer increase was not statistically significant. As expected, the chickens immunized with pcDNA3.1, pcDNA3.1/MDP1, normal saline and left unimmunized failed to demonstrate detectable HI titer (Table 4).

**Table 4:** Mean hemagglutinin inhibition (HI) results of serum samples from immunized chickens.

Group	Days post immunization				
	
	7	14	21	28	35
pcDNA3.1/H5	ND (0/9)*	0.67 ± 1.03 (2/9)	2.33 ± 0.82 (6/9)	5 ± 2.45 (9/9)	10.67 ± 4.13 (9/9)

pcDNA3.1/H5 + pcDNA3.1/MDP1	ND (0/9)	0.83 ± 0.98 (2/9)	3.33 ± 2.42 (7/9)	9.33 ± 5.46 (9/9)	13.33 ± 4.13 (9/9)

pcDNA3.1/MDP1	ND (0/9)	ND(0/9)	ND(0/9)	ND(0/9)	ND(0/9)

pcDNA3.1	ND (0/9)	ND(0/9)	ND(0/9)	ND(0/9)	ND(0/9)

Negative control	ND (0/9)	ND(0/9)	ND(0/9)	ND(0/9)	ND(0/9)

ND: Not detected

* The ratio of positive treatments to the inoculated chickens

### Reverse Transcription-Polymerase chain reaction

**Figure 2:**
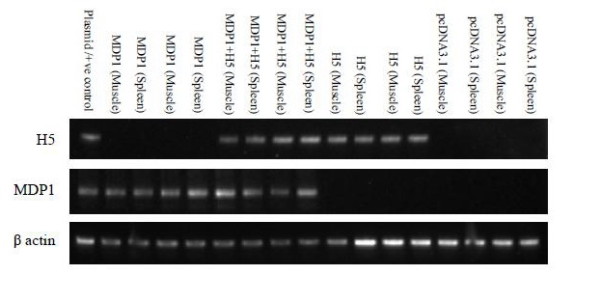
**RT-PCR analysis of H5 and MDP1 expression in different tissues obtained from chickens immunized with different DNA vaccines**. Band of the expected size (141 bp) for H5 in groups immunized with H5 and H5 + MDP1; and band of expected size (196 bp) in groups immunized with MDP1 and H5 + MDP1 was detected, respectively.

The ability of the constructed H5 and MDP1 vaccines in inducing mRNA expression for H5 and MDP1 was studied using RT-PCR following intramuscular immunization of the SPF chickens, respectively. Bands of the expected size (141 bp) indicative of H5 transcripts were detected from the spleen and muscle samples of the H5 and H5 + MDP1 immunized groups (Figure 2). Additionally, the expression of MDP1 constructed plasmid was confirmed in groups immunized with MDP1 + H5 and MDP1 alone based on the detection of bands of 196 bp in size (Figure 2).

## Discussion

Recent advances in molecular biology have raised hopes of producing more effective DNA vaccines as an alternative in preventing diseases in a much more specific and direct manner. Meanwhile, studies on animal models have provided valuable findings on the potentials of the DNA vaccine as a new option in vaccine studies and industry [5]. Prior to this study, MDP1 had been shown to be a potential DNA vaccine adjuvant in BCG, whereby it has a unique ability in blocking DNase activity, and consequently decreasing the amount of DNA necessary for vaccination [20]. Furthermore, studies have showed that MDP1 is an effective adjuvant for DNA vaccine when given separately in different plasmids through intraperitoneal and intramuscular routes of administrations [20]. In this study we showed that chickens immunized at two different sites with plasmids containing H5 and MDP1, respectively, developed higher antibody titer compared to chickens immunized with H5 alone indicating the adjuvant effect of MDP1 on AIV DNA vaccine.

The antibody responses to the H5 and H5 + MDP1 vaccine were measured using both ELISA and HI test. Meanwhile, serum samples obtained from chickens in the groups immunized with PBS, pcDNA3.1 + and pcDNA3.1/MDP1 were negative for antibody titer in both ELISA and HI test. Chickens immunized with H5 + MDP1 vaccines were able to produce detectable AIV H5 antibody 1 week earlier compared to chickens immunized with H5 vaccine alone (Table 3). The mechanisms that associated with this finding are not know where administration of MDP1 facilitate the production of antibody against H5. Furthermore, eight out of nine chickens in the H5 + MDP1 immunized group were able to develop detectable AIV H5 antibody whilst, five out of nine chickens in H5 group were able to show detectable AIV H5 antibody 35 days post immunization.

Based on HI test, the antibody production after immunization was detectable from day 14 and the production had an increasing pattern for two subsequent bleeding sessions (Table 3). The mean antibody production of the group immunized using H5 + MDP1 vaccines was slightly higher compared to the group immunized with H5 vaccine (Table 4). However, the difference was not statistically significant probably due to high standard deviation. Probably, a selection of appropriate expression plasmid construction with optimized codon usages in chickens is essential in improving the expression and regulates the delivery of the DNA vaccine for inducing significant antibody responses [21]. Furthermore, only nine chickens were used in a group in the immunization trials.

Amplification of specific regions from RNA genome was performed using RT-PCR to detect the transcription of the targeted gene in cells. Previously, Ferstl *et al. *(2004) indicated that RT-PCR is an accurate method to study the expression of desired genes in *in vivo *experiments [22]. The spleen and muscle (immunization site) samples of the chickens immunized with different DNA vaccine constructs were extracted and used as templates for PCR and RT-PCR amplifications. Agarose gel electrophoresis following RT-PCR showed successful expression of H5 mRNA for groups immunized with H5 and H5 + MDP1 vaccines (Figure 2). This finding is consistent with the results of previous studies suggesting the successful delivery and presentation of the target gene to the immune system [14,23-25]. The extracted RNA was analyzed with PCR amplification only in which no band of the expected size was detected (data not shown), indicating that the amplified product from the RT-PCR experiments were from *in vivo *transcription of the target genes.

In this study, the intramuscular immunization was performed using endotoxin-free naked H5 cloned in pcDNA3.1 +, resulted in the production of antibody against the constructed H5 DNA. This result was consistent with a study performed by Le Gall-Recule' and co-workers (2002), who found that AIV H7 cloned into an eukaryotic expression plasmid, pCMV could lead to antibody response, using different administration methods [23]. However, in another study, direct intramuscular immunization using naked plasmid did not produce the same HI titer in all the treatment, probably due to the inaccurate gene delivery system [25]. In this study, a detectable HI titer was successfully produced from the direct immunization of H5 and H5 + MDP1 vaccines in all the treatments (Table 4). Even though the mean HI titer between chicken immunized with H5 vaccine with and without MDP1 was not statistically significant, the HI titers at the different time points during the course of the experiment between the two groups were found to be significantly different and had an increasing pattern. Hence, HI test is more sensitive in detecting H5 antibody in avian compared to ELISA which is consistent with a previous study by Bulbot *et al. *[26].

In this study, the highest HI titer of 13.33 ± 4.13 was observed in chickens immunized with H5 + MDP1 vaccines on day 35 post immunization. Previous studies have shown, post immunization serum HI titre of 32 and above results in protective immunity against H5N1 influenza infection or disease in populations [26,27]. Even though we did not evaluate the constructed vaccines efficacy against viral challenge; but studies showed regardless of low antibody titers following immunization with DNA vaccine, the immunized chickens were protected against lethal challenge probably due to the cellular immune response [27-29].

## Conclusions

Our study demonstrates the potential of MDP1 as a genetic adjuvant for H5 DNA vaccine. However, chickens immunized with H5 + MDP1 vaccines developed the highest HI titer of 16 although antibody titers between chickens immunized with H5 with and without MDP1 were not statistically significant. Our future efforts will concentrate on the analysis of the cellular immune responses following the immunization using constructed H5 + MDP1 DNA vaccine.

## Competing interests

The authors declare that they have no competing interests.

## Authors' contributions

BJ designed and performed the experiments to explore the adjuvancy role of Mycobacterial DNA binding Protein 1 (MDP1) in augmenting H5 DNA vaccine in inducing specific antibody response and wrote the manuscript. ARO supervised the project and edit the manuscript. MHB and NBA co-supervised the experiments. MR participated in animal trial. SM provided the MDP1 gene and monoclonal antibody.
